# Smokeless tobacco consumption and its association with tobacco control factors in the Western Pacific Region: results from the Global Youth Tobacco Survey 2015-2019

**DOI:** 10.4178/epih.e2022103

**Published:** 2022-11-08

**Authors:** Chandrashekhar T. Sreeramareddy, Anusha Manoharan

**Affiliations:** 1 Department of Community Medicine, International Medical University, Kuala Lumpur, Malaysia; 2 Botanic Health Clinic, Ministry of Health, Selangor, Malaysia

**Keywords:** Smokeless tobacco, Adolescents, Surveys and questionnaires, Tobacco control

## Abstract

**OBJECTIVES:**

We estimated the prevalence of smokeless tobacco (ST) consumption and its associations with tobacco control factors among school-going youth in 18 Western Pacific Region (WPR) countries.

**METHODS:**

We analyzed school-based Global Youth Tobacco Survey (2014-2019) microdata from 18 WPR countries and estimated weighted prevalence rates of ST consumption, cigarette smoking, and dual use. We used multilevel binary logistic regression to examine the associations of ST consumption and dual use with demographic variables, exposure to pro-tobacco and anti-tobacco factors, national income, and MPOWER indicators.

**RESULTS:**

Data from 58,263 school-going youth were analyzed. The prevalence of past 30-day ST consumption was highest in Kiribati (42.1%), the Marshall Islands (26.1%), Micronesia (21.3%), Palau (16.0%), and Papua New Guinea (15.2%). In adjusted multilevel models, ST consumption and dual use were significantly associated with sex, age, parental smoking, pro-tobacco factors, national income, and MPOWER score. For each unit increase in score for cessation programs, we observed approximately 1.4-fold increases in the odds of youth ST consumption (adjusted odds ratio [aOR], 1.38; 95% confidence interval [CI], 1.15 to 1.66) and dual use (aOR, 1.47; 95% CI, 1.16 to 1.86). Similarly, for each unit increase in score for health-related warnings, the odds of both ST consumption (aOR, 0.47; 95% CI, 0.42 to 0.53) and dual use (aOR, 0.35; 95% CI, 0.30 to 0.42) decreased by approximately 60%.

**CONCLUSIONS:**

The prevalence of youth ST consumption was substantial in the Pacific Islands, exceeding that of cigarette smoking in some countries. Implementing MPOWER measures for ST products could help reduce ST consumption.

## INTRODUCTION

Smokeless tobacco (ST) includes various tobacco-containing products that are consumed by chewing, holding in the mouth, or sniffing [1]. ST consumption is observed among 356 million individuals in 130 nations [2], and 82% of these people live in the South-East Asia Region (SEAR) as defined by the World Health Organization (WHO) [3]. ST products are highly addictive due to their nicotine content and are known to increase the risk of head and neck cancers, oral cancer, ischemic heart disease, and stroke [4,5]. ST consumption contributes to approximately 9% of deaths and 23% of disability-adjusted life years attributable to all forms of tobacco use worldwide [4]. The SEAR has the highest prevalence of ST consumption among adults, with very little change over the past 2 decades [6]. ST use is usually initiated at a younger age than cigarette smoking [7], potentially as early as 10 years [8], which has implications for the chronic disease burden among adults [9]. Global reports based on the Global Youth Tobacco Survey (GYTS) indicate that ST consumption is prevalent among youth in more than 100 countries [10,11], including SEAR countries [12]. Nearly one-quarter of all tobacco users use ST products as a primary method of consumption [13], yet only 16 countries worldwide have implemented comprehensive bans on ST advertisement, promotion, and sponsorship [14]. ST consumption is particularly prevalent in low-income and lower-middle-income countries [10]. In many regions, increasing taxation on cigarettes has led youth to shift to ST as a less expensive alternative [15]. Overall, the widespread availability and marketing of ST products and the effect of increased cigarette taxation have likely driven increased uptake of ST among youth [16].

The Western Pacific Region (WPR) has a high prevalence of cigarette smoking, which has been projected to decline by just 12% between 2010 and 2025. In 12 WPR countries covered in the WHO global report on trends for 2000-2025, the prevalence of ST among adults ranges from 0.3% to 22.4% and 0.0% to 8.6% among men and women, respectively [17]. Most existing reports on ST use among youth are focused on South Asia [12], Nordic countries [18], and the United States [19], and to date none have focused on the WPR. Though global reports are available on the burden of ST consumption among adults and youths [6,12], the monitoring of these data in the WPR has been weak since national prevalence estimates are lacking. Furthermore, very few studies have been conducted to explore the factors associated with ST consumption. To date, a solitary study has reported pro-tobacco and anti-tobacco factors associated with ST consumption, specifically among the youth in 4 SEAR countries [12]. However, those researchers did not explore the associations of WHO MPOWER measures and the influence of parental smoking with adolescent ST consumption. Studies have shown that WHO MPOWER measures are associated with smoking [20] and electronic cigarette use [21] among youths. The WPR is the only WHO region in which all countries have ratified the Framework Convention of Tobacco Control (FCTC) [22]. The impact of the WHO FCTC applies to all forms of tobacco. Hence, it is critical to understand the tobacco control policy factors that drive ST consumption among youth to assist policymakers in the design of ST control strategies in the WPR. In this study, we provide national estimates of the prevalence of ST consumption among youth in 18 WPR countries or territories. We also assess the associations of pro-tobacco factors, anti-tobacco factors, and MPOWER measures [20] with ST consumption and dual use.

## MATERIALS AND METHODS

### Design

This study involved secondary data analyses of the most recently available GYTS data (2015-2019) from 18 WPR countries (Table 1).

### Data source

The GYTS is a self-administered, cross-sectional, nationally representative school-based survey of students aged 13 years to 15 years that is publicly available (http://nccd.cdc.gov/gtssdata/Ancillary/DataReports.aspx?CAID=2). The GYTS has a globally standardized methodology involving use of a 2-stage sample design to obtain representative samples of students in the school grades associated with 13-15 years of age. Schools are selected by sampling with probability proportional to enrollment size. Classes within the selected schools are chosen randomly. All students in the selected classes attending the school on the survey date are eligible to participate. The questionnaire covers many domains with a standard set of core questions on tobacco use, tobacco media and advertising, accessibility to tobacco products, and attitudes regarding tobacco use. It also includes a set of optional questions on ST that countries can utilize to measure and track key tobacco control indicators. The participating countries are allowed to include the optional questions or suitably adapt them to the prevailing local tobacco use behaviors. Prior to the survey, permission was received from the school authorities, and parental consent was obtained.

### Outcome variables

The main outcome measures of ST consumption included “ever tried or experimented with ST” (experimentation with any form of ST products) and “past 30-day ST consumption” (consumption of any form of ST on at least 1 day of the 30 days prior to the survey date). Current cigarette smoking was defined as having smoked cigarettes on at least 1 day of the 30 days prior to the survey. Dual use referred to ST consumption and cigarette smoking on at least 1 day of the 30 days prior to the survey date.

### Predictor variables

The independent variables included individual-level factors such as sex, age group (< 11-13, 14-15, and 16-18 years), and parental smoking status. Regarding parental smoking behavior, the question “Do your parents smoke?” had options of “no,” “both,” “father only,” “mother only,” and “I don’t know.” Responses were grouped as no/unknown, either father or mother smokes, and both parents smoke. Three anti-tobacco and 3 pro-tobacco factors were examined for potential associations with past 30-day ST consumption and dual use. The anti-tobacco factors were operationalized based on the following questions. For exposure to antitobacco messages via mass media, the question “During the past 30 days, did you see or hear any anti-tobacco media messages on TV, radio, Internet, billboards, posters, newspapers, magazines, or movies?” was asked with options of “yes” and “no.” Regarding exposure to anti-tobacco messages at events, the question “During the past 30 days, did you see or hear any anti-tobacco media messages at sports events, fairs, concerts, community events, or social gatherings?” was asked with options of “did not go to sports events, fairs, concerts, community events, or social gatherings in the past 30 days,” “yes,” and “no.” Finally, regarding being taught about the dangers of tobacco use, the question “During the past 12 months, were you taught in any of your classes about the dangers of tobacco use?” was asked with options of “yes,” “no,” and “I don’t know.” The pro-tobacco factors were also operationalized based on questions. For exposure to tobacco imagery in TV or movies, the question “During the past 30 days, did you see any people using tobacco when you watched TV, videos, or movies?” was asked with options of “did not watch TV, videos, or movies in the past 30 days,” “yes,” and “no.” Regarding exposure to tobacco advertisements, the question “During the past 30 days, did you see any advertisements or promotions for tobacco products at points of sale (such as vendors, restaurants, shops, and shopping centers)?” was asked with options of “yes” and “no.” Finally, students were asked whether they had been offered free tobacco products with the question “Has a person working for a tobacco company ever offered you a free tobacco product?”, which had potential options of “yes” and “no.”

### Country-level factors

The survey years were grouped as 2015-2017 and 2018-2019. Each country was categorized per the World Bank classification system as a low-income country (LIC), an upper middle-income country (uMIC), a lower middle-income country (lMIC), or a high-income country (HIC) based on the most recent survey year obtained from the World Bank website (https://datahelpdesk.worldbank.org/knowledgebase/articles/906519-world-bankcountry-and-lending-groups). The WHO MPOWER data were extracted from the WHO reports on the global tobacco epidemic for the closest survey year. The MPOWER indicators are focused on 6 effective strategies for fighting the global tobacco epidemic: (1) monitoring tobacco consumption and the effectiveness of preventive measures (termed “monitoring tobacco use” for this study); (2) protecting people from tobacco smoke (“smoke-free policies”); (3) offering help to quit tobacco use (“cessation programs”); (4) warning about the dangers of tobacco (“warning about dangers of tobacco use”); (5) enforcing bans on tobacco advertising, promotion, and sponsorship (“advertising bans”); and (6) raising taxes on tobacco (“taxation”). For each of these measures, a score of 1 was assigned if data were lacking entirely, if no data from 2009 onward were available, or if available data were not both recent and representative of the national population. Scores of 2 to 4 (for M) and 2 to 5 (for P, O, W, E, and R) represent a scale from the weakest to the strongest level of tobacco control policy in the relevant country. A score was ascertained for each of the 6 dimensions, and the 6 scores were summed to obtain the MPOWER score. The highest possible MPOWER score was 29.

### Statistical analysis

For each country, we estimated the weighted prevalence (%) and 95% confidence intervals (CIs) of past 30-day cigarette smoking, “ever tried ST,” past 30-day ST consumption, and dual use. Because the GYTS involves a multistage sampling process, we used survey weights to adjust for the school, class, and student selected. After pooling the data from the 18 countries, each survey participant (or student) is nested within the school, and each school is nested within the country. To account for this, multilevel binary logistic regression models with a random intercept at the school (the first level) and country (the second level) were used. To explore the individual factors, pro-tobacco and anti-tobacco factors, and MPOWER scores [20] associated with past 30-day ST consumption and dual use, adjusted odds ratios (aORs), 95% CIs, and random-effects parameters were estimated, and the likelihood ratio test was conducted. The individual variables included age, sex, current cigarette smoking status, and exposure to pro-tobacco and anti-tobacco factors, while the country-level variables were survey year, World Bank income group, and MPOWER score. Three models were developed. Model 1 included the univariate analyses, model 2 incorporated demographic factors along with exposure to pro-tobacco and anti-tobacco factors, and model 3 was the full model after the inclusion of country-level variables. Data on parental smoking status were collected in only 12 countries, and this variable was therefore included in a separate multilevel model. Additionally, we explored the associations of each WHO-MPOWER measure with past 30-day ST consumption and dual use, with adjustment for age, sex, survey year, and World Bank income category. All analyses were performed using Stata version 11 (StataCorp., College Station, TX, USA).

### Ethics statement

For the GYTS, ethical approval was obtained from the United States Centers for Disease Control and Prevention in Atlanta, GA, as well as the implementing institutions in each country. Consent for participation was sought from school authorities and parents. Because de-identified publicly available secondary data were used to prepare this report, separate ethical approval was not required for this study.

## RESULTS

Table 1 shows survey characteristics and MPOWER measures in the 18 WPR countries analyzed. In these countries, a total of 58,263 youths were surveyed between 2015 and 2019, of whom 31,414 were aged 13-15 years. The response rate was > 70% in 13 countries. Lao People’s Democratic Republic (Lao PDR), Mongolia, and Palau had response rates of > 90%, while the lowest rate was associated with the Cook Islands (56.6%). In most countries, approximately half of the sample surveyed was 13-15 years old. Three countries were HICs (Brunei, Guam, and Macau), 8 were lMICs (Cambodia, Kiribati, Lao PDR, Micronesia, Papua New Guinea, the Philippines, Samoa, and Vanuatu), and 7 were uMICs (the Cook Islands, Fiji, the Marshall Islands, Mongolia, Niue, Palau, and Tuvalu). MPOWER scores ranged from 18 (Papua New Guinea) to 24 (the Cook Islands, Mongolia, and the Philippines).

### Prevalence of smokeless tobacco consumption and cigarette smoking

Table 2 provides national prevalence estimates of ST consumption and cigarette smoking. The prevalence of “ever trying” or experimenting with ST products was over 20% in 7 countries: Kiribati (51.1%), Mongolia (41.6%), the Marshall Islands (37.5%), Micronesia (32.1%), Palau (26.5%), Papua New Guinea (27.2%), and Guam (21.7%), while in 11 countries it was 5.7% (Niue) or lower. Past 30-day ST consumption among youth was < 5.0% in 10 countries; in contrast, Kiribati (42.1%) had the highest prevalence followed by the Marshall Islands (26.1%) and Micronesia (21.3%). The prevalence of either cigarette smoking or ST consumption was at least 10% in all countries except Brunei (8.1%), Macau (5.7%), and Cambodia (1.9%). Consumption of either type of tobacco was highest in Kiribati (30.4%), followed by Palau (29.0%) and Papua New Guinea (24.8%). The prevalence of dual use was < 1.0% in 9 countries, while it was highest in Kiribati (17.0%), the Marshall Islands (13.4%) and Micronesia (12.0%). The prevalence of cigarette smoking ranged from 0.9% in Cambodia to 31.8% in Palau. The past 30-day cigarette smoking prevalence was > 20% in 6 countries (Kiribati, the Marshall Islands, Micronesia, Palau, Papua New Guinea, and Vanuatu). Estimates by sex are shown in Supplementary Materials 1 and 2. Tobacco use prevalence rates were higher among boys than girls. However, in countries where ST consumption was high, the sex differentials were narrow for the 30-day ST prevalence and were even narrower for the past 30-day prevalence of cigarette smoking. In countries with lower ST consumption, the sex differentials were much wider, with higher rates among boys than girls (Supplementary Material 3).

### Factors associated with smokeless tobacco and dual use

The results of the multilevel fixed effects regression analyses are shown in Tables 3-5. In all models, age and sex were associated with past 30-day ST consumption and dual use. Girls had lower odds than boys, while youth aged 14-15 years and 16-18 years had higher odds of ST consumption and dual use than youth aged 11-13 years. In model 1, all 3 anti-tobacco factors were significantly associated with ST consumption. However, exposure to anti-tobacco messages at events and being taught about the dangers of tobacco were not significantly associated with ST consumption in models 2 or 3. For dual use, all 3 anti-tobacco factors were found to be significant in model 3. The direction of association and effect size for country income and MPOWER score was reversed in model 3 compared to model 1. Data on parental smoking were available for only 12 countries. In the additional models, the inclusion of parental smoking status attenuated the associations of both ST consumption and dual use with MPOWER scores. Youth whose parents were smokers had relatively high odds of ST consumption and dual use. In model 1, the odds of ST consumption and dual use were significantly higher if one or both parents were cigarette smokers than if neither parent smoked cigarettes. In model 3, the association remained significant, but the effect size was smaller. If both parents were cigarette smokers, the odds of ST consumption and dual use were approximately 1.7 times (aOR, 1.68; 95% CI, 1.44 to 1.97) and 2.2 times (aOR, 2.15; 95% CI, 1.74 to 2.65) higher, respectively, than if the parents were non-smokers (Supplementary Material 3).

Exposure to anti-tobacco information through mass media was significantly associated with ST consumption. However, exposure to anti-tobacco information at community events and being taught in the classroom were significantly associated with dual use. Among the pro-tobacco factors, exposure to tobacco advertisement and promotion, as well as offers of free tobacco products, were associated with both ST and dual use. Youth who were exposed to tobacco advertisements and promotions had approximately 1.2 (aOR, 1.18; 95% CI, 1.08 to 1.29) and 1.5 (aOR, 1.47; 95% CI, 1.31 to 1.67) times higher odds of ST consumption and dual use, respectively. Youth who were offered free tobacco products had approximately twice (aOR, 1.95; 95% CI, 1.74 to 2.18) and thrice (aOR, 2.84; 95% CI, 2.43 to 3.23) higher odds of being ST users and dual users, respectively.

At the national level, survey year, income category, and MPOWER score were associated with both ST consumption and dual use. Youth in HICs or uMICs had lower odds of ST consumption and dual use than youth living in lMICs. Each unit increase in MPOWER score carried nearly 25% lower odds of ST consumption (aOR, 0.76; 95% CI, 0.70 to 0.81) and nearly 50% lower odds of dual use (aOR, 0.55; 95% CI, 0.50 to 0.60) (Supplementary Material 3). Youth from HICs had 0.11 times the odds of both ST consumption (aOR, 0.11; 95% CI, 0.08 to 0.15) (Table 3) and dual use (aOR, 0.11; 95% CI, 0.07 to 0.17) compared to those from LMICs (Table 4).

Among the MPOWER indicators, in the full model after adjustment for age, sex, survey year, and income group, both cessation programs and warnings about the dangers of tobacco use were associated with both ST consumption and dual use. For each unit increase in the score for cessation programs, we observed approximately 1.4-fold increases in the odds of ST consumption (aOR, 1.38; 95% CI, 1.15 to 1.66) and dual use (aOR, 1.47; 95% CI, 1.16 to 1.86) among the youth. Similarly, for each unit increase in the score about warnings of the health effects of tobacco use, the odds of both ST consumption (aOR, 0.47; 95% CI, 0.42 to 0.53) and dual use (aOR, 0.35; 95% CI, 0.30 to 0.42) among the youth decreased by approximately 60%.

## DISCUSSION

These results show that the past 30-day prevalence of ST consumption was higher than 10% in one-third of the 18 WPR countries studied, and the prevalence of cigarette smoking was higher than 20% in one-third of the countries. The overall past 30-day prevalence for either type of tobacco product was > 10% in 15 countries. In 3 countries, the prevalence of dual use was > 10%. ST consumption was relatively high among Pacific Island nations. In some of them, the prevalence of ST consumption was higher than that of cigarette smoking, and the prevalence among girls was nearly the same as that among boys. Multilevel regression analyses showed that male sex, older age, parental smoking, and pro-tobacco factors were positively associated with past 30-day ST consumption and dual use. MPOWER score was negatively associated with both ST consumption and dual use (after adjustment for smoking status), while national income was negatively associated. Among the 6 MPOWER measures, the score for cessation programs was positively associated and the score for warnings about the dangers of tobacco use was negatively associated with ST use and dual use. The score for taxation was positively associated with dual use only.

The strength of our report was its inclusion of national prevalence estimates based on representative samples of school-going youth, which are comparable across countries and over time because GYTS protocols and questionnaires are standardized. We tested the associations of ST consumption with pro-tobacco and anti-tobacco factors, as well as 6 MPOWER measures. Nevertheless, this report has limitations inherent in the GYTS survey design. Since the GYTS is a school-based survey, the estimates do not represent non-school-going children. Additionally, self-reported tobacco use is known to be underreported in school-based surveys, leading to an underestimation of prevalence. The identified associations should also be interpreted in the context of the dynamic nature of tobacco use behaviors under the changing tobacco control environments present in the 17 countries studied in 2014-2019. The cross-sectional design of the GYTS also limits temporal causal interpretation between tobacco control factors and ST consumption. Countries with stronger anti-tobacco political commitment, such as Brunei, may have had stronger regulatory policies and implementation. Fewer regulations and weaker implementation are also expected in countries where tobacco use is less of a problem, such as Cambodia. Additionally, the prevalence estimates are influenced by the marketing strategies of tobacco companies, data about which were unavailable in the GYTS. The tobacco control factors tested in the multilevel analyses may not specifically refer to ST products, since most questions in the GYTS generally refer to cigarette smoking. Finally, school-level policy data about cigarette smoking and/or ST consumption were not available.

The estimates for both trial and 30-day ST consumption were relatively high among island nations, most of which were lower income (except) and had smaller sex differentials than the other countries; this aligned with previous studies from the GYTS [3,7] and a global report [10]. Curiosity about tobacco products and subsequent experimentation has been shown to result in regular smoking behavior [23], which explains the finding that dual use was higher in countries with higher ST consumption rates. South Asia is known to have the highest rates of ST consumption [12]. Nevertheless, our study shows that in some island nations of the WPR, ST consumption rates were alarming, while rates of cigarette smoking were higher in other WPR countries. Considering both types of products, the burden of tobacco use among youth was found to be substantial in most WPR countries. The increasing rates of ST consumption among older respondents align with reports from other countries [24,25]. This finding also supports the early age at initiation of tobacco use [26]. The relatively high prevalence in some island nations was reported in earlier studies as well as in the Global Burden of Disease report [4,7,26]. The high rate of ST consumption in some WPR countries has been attributed to the culturally acceptable presence of locally available ST products, which are easily accessible for purchase at a cheaper price. Moreover, ST products are not usually covered by tobacco control laws and hence are unregulated. ST products are typically manufactured and marketed by unorganized sectors in developing countries and are known to thrive under weak tobacco control policy environments [27,28].

A conducive home environment in which parents smoke promotes both smoking and other tobacco usage (such as ST); this is consistent with a GYTS report focused on Africa [29]. The associations between exposure to tobacco advertisements, being offered an ST product, receiving information about ST products, and dual usage among youths [12,30] are also consistent with the literature. The lack of association noted for exposure to anti-tobacco messages in mass media, exposure through events, being taught about the dangers of smoking, and exposure to tobacco imagery is consistent with a GYTS-based report from 4 SEAR countries [12]. These results indicate that anti-tobacco messages generally targeting adults may not be reaching school-going youth. We tested the association between ST and dual use with MPOWER scores as well as the 6 individual MPOWER strategies. When parental smoking status was not included due to the lack of available data in 6 countries, MPOWER score was positively associated with ST consumption and dual use. However, in adjusted models that included parental smoking status, the MPOWER score was protective for ST consumption and dual use. Our results align with ecological analyses finding that MPOWER strategies have reduced the prevalence of smoking across the globe [31,32]. Additionally, our results suggest that even under strict tobacco control measures, parents’ smoking behaviors are influential on youth tobacco use behavior. Since MPOWER score is a composite index, we disentangled the MPOWER score by the inclusion of individual MPOWER strategies in the regression models to test the association of individual strategies on ST consumption and dual use. The results showed that cessation programs were positively associated with ST and dual use, implying that cigarette cessation programs may have driven the compensatory uptake of ST. Chan et al. [21] showed that increased taxation was associated with electronic cigarette use among youth. The negative association with warnings indicates the success of pictorial warnings in deterring tobacco use [33-35].

### Policy implications

ST consumption must be monitored more closely in the island nations of the WPR. Tobacco control policies should cover ST products, specifically health warnings and assistance to quit ST product consumption. ST products should also be covered under taxation policies to deter the uptake of less expensive ST tobacco products. Specific regulatory policies could be formulated to tackle the rising prevalence of ST products. Tailored school-based educational campaigns should be developed and implemented to dispel myths related to ST [14].

In conclusion, the prevalence of cigarette, ST, and dual usage among youth remains resilient in the WPR, a region in which all countries are signatories of the WHO FCTC. Strengthening and enforcement of comprehensive regulations on ST products are needed in the Pacific Islands nations. The results suggest that health warnings on ST products are also necessary, and cessation programs should be incorporated for ST consumers.

## Figures and Tables

**Table 1.** GYTS survey characteristics and WHO MPOWER scores for 18 countries of the WHO WPR

Country	Year of survey	Income category	Response rate (%)	Survey sample	Sample aged 13-15 yr	Monitoring tobacco use	Smoke-free policies	Cessation programs	Warnings about the dangers of tobacco	Advertising bans	Taxation	WHO MPOWER score^1^
Brunei Darussalam^2^	2019	HIC	67.7	2,674	1,549	4	5	4	5	4	-	22
Cambodia	2016	lMIC	81.0	3,716	1,866	4	5	3	5	4	3	20
Cook Islands	2016	uMIC	56.6	614	334	4	4	4	4	4	4	24
Fiji	2016	uMIC	80.7	3,697	1,639	3	3	4	5	4	3	22
Guam^3^	2017	HIC	70.7	2,506	1,129	-	-	-	-	-	-	-
Kiribati	2018	lMIC	71.2	2,622	1,190	2	3	4	3	5	3	20
Lao PDR	2016	lMIC	98.0	6,550	3,930	4	5	2	5	4	2	22
Macau, China	2015	HIC	76.6	1,907	1,182	3	2	4	3	4	4	20
Marshall Islands	2016	uMIC	83.8	3,522	1,434	2	5	4	2	2	4	19
Micronesia	2019	lMIC	87.1	6,660	3,398	2	4	4	2	4	3	19
Mongolia	2019	uMIC	92.1	4,146	3,630	4	3	4	5	5	3	24
Niue	2019	uMIC	77.3	163	68	1	5	4	3	5	5	23
Palau	2017	uMIC	92.7	1,484	637	4	4	4	2	4	4	22
Papua New Guinea	2016	lMIC	60.0	2,301	1,400	2	5	2	2	4	3	18
Philippines	2015	lMIC	83.5	10,602	6,670	4	3	4	5	4	4	24
Samoa	2017	lMIC	61.9	2,076	1,057	2	4	4	5	4	4	23
Tuvalu	2018	uMIC	89.9	764	424	3	3	3	3	5	3	20
Vanuatu	2017	lMIC	64.5	2,257	1,067	3	2	3	5	5	4	22

GYTS, Global Youth Tobacco Survey; WHO, World Health Organization; WPR, Western Pacific Region; HIC, high-income country; lMIC, lower middle-income country; uMIC, upper middle-income country; Lao PDR, Lao People’s Democratic Republic.

1 The individual maximum scores are 4 for M and 5 for P, O, W, E, and R; The total maximum MPOWER score is 29.

2 Taxation is not applicable since the sale of tobacco is banned in Brunei.

3 MPOWER data were not available for Guam.

**Table 2.** ST use, cigarette smoking, and dual use among youth in 18 countries of the WHO WPR

Country	Ever tried or experimented with ST	ST consumption in the past 30 day	Either cigarette smoking or ST consumption in the past 30 day	Dual use (both cigarette smoking and ST consumption) in the past 30 day	Cigarette smoking in the past 30 day
Brunei Darussalam	2.0 (1.4, 2.5)	1.0 (0.5, 1.5)	8.1 (5.5, 10.6)	0.1 (0.0, 0.3)	7.7 (5.2, 10.1)
Cambodia	3.0 (2.5, 3.6)	1.1 (0.8, 1.4)	1.9 (1.2, 2.6)	0.0 (0.0, 0.1)	0.9 (0.4, 1.5)
Cook Islands	5.5 (5.5, 5.5)	3.8 (3.8, 3.8)	17.1 (17.1, 17.1)	2.8 (2.8, 2.8)	19.1 (19.1, 19.1)
Fiji	4.5 (3.4, 5.7)	2.4 (1.5, 3.3)	12.2 (9.1, 15.3)	0.6 (0.3, 0.9)	12.1 (8.9, 15.3)
Guam	21.7 (19.4, 24.1)	11.1 (9.3, 12.8)	10.5 (9.0, 12.0)	4.7 (3.5, 5.9)	11.0 (9.4, 12.6)
Kiribati	51.1 (47.2, 55.0)	42.1 (38.5, 45.7)	30.4 (27.7, 33.2)	17.0 (14.2, 19.8)	24.1 (21.0, 27.2)
Lao PDR	5.7 (4.8, 6.6)	3.7 (3.0, 4.4)	10.0 (8.4, 11.7)	0.7 (0.4, 0.9)	7.9 (6.4, 9.5)
Macau, China	4.0 (3.0, 5.0)	1.8 (1.2, 2.4)	5.7 (3.0, 8.4)	0.4 (0.1, 0.7)	4.8 (2.0, 7.5)
Marshall Islands	37.5 (34.9, 40.1)	26.1 (23.7, 28.5)	19.6 (17.7, 21.4)	13.4 (11.5, 15.3)	22.0 (19.4, 24.7)
Micronesia	32.1 (30.1, 34.2)	21.3 (19.2, 23.4)	19.7 (18.2, 21.2)	12.0 (10.6, 13.4)	23.6 (21.7, 25.6)
Mongolia	41.6 (37.1, 46.0)	8.1 (6.6, 9.7)	11.0 (9.2, 12.8)	0.8 (0.5, 1.2)	4.7 (3.5, 5.9)
Niue	5.7 (1.2, 10.2)	2.5 (-0.1, 5.1)	11.3 (5.8, 16.9)	1.3 (-0.4, 2.9)	11.8 (5.6, 17.9)
Palau	26.5 (23.4, 29.6)	16.0 (13.4, 18.6)	29.0 (25.8, 32.3)	8.7 (6.9, 10.6)	31.8 (28.3, 35.3)
Papua New Guinea	27.2 (22.9, 31.5)	15.2 (11.5, 19.0)	24.8 (18.1, 31.6)	6.8 (4.7, 8.8)	24.7 (17.9, 31.5)
Philippines	4.9 (3.5, 6.3)	2.5 (1.8, 3.2)	14.2 (12.5, 15.9)	0.8 (0.6, 1.0)	14.7 (12.8, 16.6)
Samoa	5.2 (4.2, 6.3)	2.6 (1.7, 3.5)	13.6 (10.6, 16.6)	0.4 (0.2, 0.7)	12.6 (9.6, 15.6)
Tuvalu	5.6 (3.2, 7.9)	3.2 (1.9, 4.4)	17.0 (13.4, 20.5)	0.7 (0.0, 1.4)	15.9 (12.2, 19.5)
Vanuatu	9.1 (7.3, 10.8)	5.3 (3.9, 6.6)	21.4 (17.7, 25.1)	2.1 (1.2, 3.0)	21.2 (17.1, 25.4)

Values are presented as weighted prevalence (95% confidence interval). ST, smokeless tobacco; WHO, World Health Organization; WPR, Western Pacific Region; Lao PDR, Lao People’s Democratic Republic.

**Table 3.** Individual and country-level factors associated with ST use among youth in 17 countries of the WHO WPR^1^

Variables			Model 1		Model 2		Model 3	
			Crude	p-value	Adjusted	p-value	Adjusted	p-value
Individual factors								
	Sex							
		Male	1.00 (reference)		1.00 (reference)		1.00 (reference)	
		Female	0.69 (0.55, 0.86)	0.001	0.79 (0.73, 0.85)	<0.001	0.77 (0.71, 0.83)	<0.001
	Age (yr)							
		11-13	1.00 (reference)		1.00 (reference)		1.00 (reference)	
		14-15	0.93 (0.61, 1.44)	0.754	1.12 (1.00, 1.26)	0.057	1.11 (.99, 1.25)	0.084
		16-18	1.16 (0.71, 1.89)	0.563	1.60 (1.41, 1.82	<0.001	1.58 (1.39, 1.79)	<0.001
	Cigarette smoking							
		No	1.00 (reference)		1.00 (reference)		1.00 (reference)	
		Yes	4.89 (3.89, 6.15)	<0.001	5.25 (4.79, 5.76)	<0.001	4.75 (4.33, 5.22)	<0.001
	Parental smoking							
		None/unknown	1.00 (reference)					
		Either parent	1.71 (1.28, 2.28)	<0.001				
		Both parents	3.58 (2.45, 5.24)	<0.001				
Pro-tobacco and anti-tobacco factors								
	Exposure to anti-tobacco messages via mass media							
		No	1.00 (reference)		1.00 (reference)		1.00 (reference)	
		Yes	0.68 (0.50, 0.93)	0.014	1.12 (1.03, 1.22)	0.011	1.14 (1.04, 1.24)	0.004
	Exposure to anti-tobacco messages at events							
		No/did not attend	1.00 (reference)		1.00 (reference)		1.00 (reference)	
		Yes	1.46 (1.18, 1.81)	0.001	1.06 (0.98, 1.16)	0.163	1.06 (0.97, 1.15)	0.205
	Taught about the dangers of tobacco use							
		No/don’t know	1.00 (reference)		1.00 (reference)		1.00 (reference)	
		Yes	0.63 (0.50, 0.80)	<0.001	1.07 (0.98, 1.16)	0.109	1.07 (0.98, 1.17)	0.111
	Exposure to tobacco imagery in TV/movies							
		No/did not watch	1.00 (reference)		1.00 (reference)		1.00 (reference)	
		Yes	0.69 (0.56, 0.87)	0.001	1.07 (0.98, 1.17)	0.117	1.09 (1.0, 1.29)	0.052
	Exposure to tobacco advertisements							
		No	1.00 (reference)		1.00 (reference)		1.00 (reference)	
		Yes	1.37 (1.06, 1.78)	0.017	1.18 (1.08, 1.28)	<0.001	1.18 (1.08, 1.29)	<0.001
	Offered free tobacco products							
		No	1.00 (reference)		1.00 (reference)		1.00 (reference)	
		Yes	3.33 (2.40, 4.61)	<0.001	2.01 (1.79, 2.24)	<0.001	1.95 (1.74, 2.18)	<0.001
Country-level factors								
	Year of survey							
		2015-17	1.00 (reference)		-		1.00 (reference)	
		2018-19	3.03 (2.41, 3.81)	<0.001	-		3.14 (2.49, 3.96)	<0.001
	National income							
		Lower middle	1.00 (reference)		-		1.00 (reference)	
		Upper middle	1.09 (0.81, 1.44)	0.540	-		0.34 (0.25, 0.46)	<0.001
		High	1.18 (0.91, 1.54)	0.216	-		0.11 (0.08, 0.15)	<0.001
	MPOWER score		0.81 (0.71, 0.92)	0.001	-		1.26 (1.22, 1.32)	<0.001

Values are presented as odds ratio (95% confidence interval). ST, smokeless tobacco; WHO, World Health Organization; WPR, Western Pacific Region; Lao PDR, Lao People’s Democratic Republic.

1 Data on parental smoking were unavailable for 5 countries (Brunei, Lao PDR, the Marshall Islands, Palau, and the Philippines); MPOWER scores were unavailable for Guam.

**Table 4.** Individual and country-level factors associated with dual use among youth in 17 countries of the WHO WPR^1^

Variables			Model 1		Model 2		Model 3	
			Crude	p-value	Adjusted	p-value	Adjusted	p-value
Individual factors								
	Sex							
		Male	1.00 (reference)		1.00 (reference)		1.00 (reference)	
		Female	0.31 (0.18, 0.53)	<0.001	0.85 (0.72, 1.00)	0.049	0.41 (0.36, 0.46)	<0.001
	Age (yr)							
		11-13	1.00 (reference)		1.00 (reference)		1.00 (reference)	
		14-15	1.34 (0.73, 2.46)	0.350	1.45 (1.11, 1.90)	0.007	2.05 (1.70, 2.52)	<0.001
		16-18	2.18 (1.17, 4.06)	0.010	2.04 (1.56, 2.67)	<0.001	3.80 (3.10, 4.67)	<0.001
	Parental smoking^2^							
		None/don’t know	1.00 (reference)		-		-	
		Either parent	2.07 (1.27, 3.37)	<0.001	-		-	
		Both parents	6.13 (2.75, 13.65)	<0.001	-		-	
Pro-tobacco and anti-tobacco factors								
	Exposure to anti-tobacco messages via mass media							
		No	1.00 (reference)		1.00 (reference)		1.00 (reference)	
		Yes	0.77 (0.45, 1.33)	0.350	1.27 (1.07, 1.50)	0.006	1.20 (1.05, 1.36)	0.006
	Exposure to anti-tobacco messages at events							
		No/did not attend	1.00 (reference)		1.00 (reference)		1.00 (reference)	
		Yes	2.44 (1.79, 3.34)	0.001	0.96 (0.82, 1.13)	0.610	1.37 (1.22, 1.55)	<0.001
	Taught about the dangers of tobacco use							
		No/don’t know	1.00 (reference)		1.00 (reference)		1.00 (reference)	
		Yes	0.61 (0.39, 0.95)	0.030	1.14 (0.97, 1.34)	0.120	1.17 (1.03, 1.33)	0.013
	Exposure to tobacco imagery in TV/movies							
		No/did not watch	1.00 (reference)		1.00 (reference)		1.00 (reference)	
		Yes	0.71 (0.49, 1.02)	0.060	1.03 (0.87, 1.22)	0.730	1.09 (0.97, 1.25)	0.160
	Exposure to tobacco advertisements							
		No	1.00 (reference)				1.00 (reference)	
		Yes	2.20 (1.44, 3.35)	<0.001	1.27 (1.08, 1.49)	0.004	1.47 (1.31, 1.67)	<0.001
	Offered free tobacco products							
		No	1.00 (reference)		1.00 (reference)		1.00 (reference)	
		Yes	5.74 (3.62, 9.12)	<0.001	1.94 (1.60, 2.35)	<0.001	2.84 (2.43, 3.23)	<0.001
Country-level factors								
	Year of survey							
		2015-17	1.00 (reference)		-		1.00 (reference)	
		2018-19	2.17 (1.60, 2.94)	<0.001	-		5.07 (3.59, 7.15)	<0.001
	National income							
		Lower middle	1.00 (reference)		-		1.00 (reference)	
		Upper middle	1.07 (0.76, 1.49)	0.710	-		0.27 (0.18, 0.42)	<0.001
		High	1.36 (0.99, 1.87)	0.050	-		0.11 (0.07, 0.17)	<0.001
	MPOWER score		0.79 (0.69, 0.92)	<0.001	-		1.31 (1.24, 1.39)	<0.001

Values are presented as odds ratio (95% confidence interval). WHO, World Health Organization; WPR, Western Pacific Region; Lao PDR, Lao People’s Democratic Republic.

1 Data on parental smoking were unavailable for 5 countries (Brunei, Lao PDR, the Marshall Islands, Palau, and the Philippines); MPOWER scores were unavailable for Guam.

2 Parental smoking status was included in a separate multilevel model.

**Table 5.** Associations between WHO-MPOWER indicators and other factors associated with past 30-day ST tobacco use and dual use (both ST use and cigarette smoking) in 17 countries of the WHO WPR^1^

Variables		ST use				Dual use			
		Univariate		Multivariate		Univariate		Multivariate	
		Crude	p-value	Adjusted	p-value	Crude	p-value	Adjusted	p-value
Sex									
	Mal	1.00 (reference)		1.00 (reference)		1.00 (reference)		1.00 (reference)	
	Female	0.69 (0.55, 0.86)	0.001	0.60 (0.56, 0.65)	<0.001	0.31 (0.18, 0.53)	<0.001	0.37 (0.33, 0.41)	<0.001
Age (yr)									
	11-13	1.00 (reference)		1.00 (reference)		1.00 (reference)		1.00 (reference)	
	14-15	0.93 (0.61, 1.44)	0.754	1.28 (1.15, 1.41)	<0.001	1.34 (0.73, 2.46)	0.350	2.19 (1.83, 2.61)	<0.001
	16-18	1.16 (0.71, 1.89)	0.563	2.08 (1.87, 2.32)	<0.001	2.18 (1.17, 4.06)	0.010	4.24 (3.56, 5.05)	<0.001
Year of survey									
	2015-17	1.00 (reference)		1.00 (reference)		1.00 (reference)		1.00 (reference)	
	2018-19	3.03 (2.41, 3.81)	<0.001	1.25 (0.92, 1.70)	0.158	2.17 (1.60, 2.94)	0.001	1.21 (0.83, 1.77)	0.324
National income									
	Lower middle	1.00 (reference)		1.00 (reference)		1.00 (reference)		1.00 (reference)	
	Upper middle	1.09 (0.81, 1.44)	0.540	0.81 (0.63, 1.03)	0.084	1.07 (0.76, 1.49)	0.710	0.79 (0.58, 1.08)	0.138
	High	1.18 (0.91, 1.54)	0.216	0.27 (0.18, 0.41)	<0.001	1.36 (0.99, 1.87)	0.050	0.25 (0.15, 0.42)	<0.001
MPOWER measure									
	Monitoring tobacco use	0.38 (0.31, 0.46)	<0.001	1.10 (0.94, 1.29)	0.237	0.31 (0.26, 0.38)	<0.001	1.03 (0.85, 1.25)	0.733
	Smoke-free policies	1.39 (1.10, 1.75)	0.006	0.98 (0.84, 1.15	0.835	1.41 (1.08, 1.85)	0.012	1.04 (0.86, 1.27)	0.664
	Cessation programs	0.56 (0.43, 0.72)	<0.001	1.38 (1.15, 1.66)	<0.001	0.52 (0.39, 0.69)	<0.001	1.47 (1.16, 1.86)	0.001
	Warnings about the dangers of tobacco	0.51 (0.45, 0.58)	<0.001	0.47 (0.42, 0.53)	<0.001	0.45 (0.40, 0.51)	<0.001	0.35 (0.30, 0.42)	<0.001
	Advertising bans	3.14 (2.44, 4.03)	<0.001	1.06 (0.79, 1.41)	0.693	1.48 (1.00, 2.20)	0.052	1.31 (0.92, 1.86)	0.133
	Taxation	0.62 (0.48, 0.79)	<0.001	1.08 (0.86, 1.34	0.518	0.69 (0.52, 0.91)	0.009	1.46 (1.11, 1.92)	0.007

Values are presented as odds ratio (95% confidence interval). ST, smokeless tobacco; WHO, World Health Organization; WPR, Western Pacific Region.

1 Guam was not included in the analyses since MPOWER scores were unavailable for Guam.

## SUPPLEMENTARY MATERIALS

Supplementary Material 1. Sex-wise weighted prevalence estimates and 95% CI of smokeless tobacco use among the youth in 18 countries in the Western Pacific region

Supplementary Material 2 sex-wise weighted prevalence estimates of cigarette smoking or ST use (either) or dual-use use among the youth in 18 countries in the Western Pacific region

Supplementary Material 3 Individual and country-level factors associated with ST use and dual-use by multi-level analyses after inclusion of parental smoking status in 12 countries of WHO WPR
