# Supplementary material for: Smokeless tobacco consumption and its association with tobacco control factors in the Western Pacific Region: results from the Global Youth Tobacco Survey 2015-2019
Source: Epidemiol Health. 2022 Nov 8;44:e2022103. doi: 10.4178/epih.e2022103 (PMC10185971; doi:10.4178/epih.e2022103)
Supplement: Supplementary Material 1. — Sex-wise weighted prevalence estimates and 95% CI of smokeless tobacco use among the youth in 18 countries in the Western Pacific region [file epih-44-e2022103-Supplementary-1.docx]

**Supplementary Material 1.** Sex-wise weighted prevalence estimates and 95% CI of smokeless tobacco use among the youth in 18 countries in the Western Pacific region

|  | Boys | Girls | Boys | Girls |
| --- | --- | --- | --- | --- |
|  | Ever tried or experimented ST | | Used ST past 30-days | |
| **Brunei Darussalam** | 2.5 (1.6, 3.4) | 1.4 (0.8, 2.1) | 1.6 (0.8, 2.4) | 0.4 (0.1, 0.7) |
| **Cambodia** | 3.2 (2.3, 4.1) | 2.8 (2.0, 3.6) | 1.3 (0.8, 1.9) | 0.9 (0.5, 1.3) |
| **Cook Islands** | 7.5 (7.5, 7.5) | 3.7 (3.7, 3.7) | 4.6 (4.6, 4.6) | 3.1 (3.1, 3.1) |
| **Fiji** | 6.3 (4.9, 7.6) | 2.6 (1.7, 3.5) | 3.1 (1.9, 4.4) | 1.6 (0.8, 2.3) |
| **Guam** | 23.1 (19.9, 26.2) | 20.1 (17.3, 22.8) | 12.9 (10.4, 15.5) | 8.8 (7.0, 10.7) |
| **Kiribati** | 54.5 (49.7, 59.2) | 48.1 (43.9, 52.2) | 45.6 (41.6, 49.6) | 39.2 (35.0, 43.4) |
| **Lao PDR** | 6.8 (5.4, 8.3) | 4.5 (3.8, 5.2) | 4.4 (3.3, 5.6) | 2.9 (2.3, 3.4) |
| **Macao, China** | 3.9 (2.8, 5.0) | 4.0 (2.3, 5.8) | 1.9 (1.2, 2.6) | 1.7 (0.7, 2.6) |
| **Marshall Islands** | 47.6 (43.8, 51.4) | 29.5 (26.4, 32.6) | 34.3 (30.7, 37.9) | 19.7 (17.2, 22.2) |
| **Micronesia,** | 36.7 (33.9, 39.5) | 28.3 (25.7, 31.0) | 26.2 (23.6, 28.8) | 17.4 (15.1, 19.6) |
| **Mongolia** | 47.9 (42.7, 53.2) | 35.4 (31.5, 39.3) | 11.8 (9.1, 14.5) | 4.5 (3.5, 5.5) |
| **Niue** | 7.2 (0.3, 14.0) | 4.6 (-0.1, 9.3) | 2.9 (-0.8, 6.6) | 2.2 (-1.9, 6.2) |
| **Palau** | 25.4 (20.9, 29.9) | 27.4 (23.8, 31.0) | 15.2 (11.4, 19.1) | 16.7 (13.8, 19.6) |
| **Papua New Guinea** | 26.3 (21.6, 30.9) | 27.0 (21.4, 32.6) | 15.7 (11.8, 19.5) | 14.7 (9.1, 20.3) |
| **Philippines -** | 5.8 (4.3, 7.3) | 4.0 (2.6, 5.5) | 3.0 (2.3, 3.8) | 2.0 (1.2, 2.8) |
| **Samoa** | 5.7 (3.9, 7.6) | 4.6 (3.6, 5.5) | 3.4 (2.0, 4.8) | 1.7 (1.1, 2.3) |
| **Tuvalu** | 5.6 (2.4, 8.8) | 5.6 (2.2, 8.9) | 4.1 (1.8, 6.4) | 2.5 (1.2, 3.8) |
| **Vanuatu** | 9.5 (7.1, 11.9) | 8.5 (6.1, 10.9) | 5.5 (3.7, 7.3) | 4.9 (3.4, 6.4) |
