# Supplementary material for: Smokeless tobacco consumption and its association with tobacco control factors in the Western Pacific Region: results from the Global Youth Tobacco Survey 2015-2019
Source: Epidemiol Health. 2022 Nov 8;44:e2022103. doi: 10.4178/epih.e2022103 (PMC10185971; doi:10.4178/epih.e2022103)
Supplement: Supplementary Material 2 — sex-wise weighted prevalence estimates of cigarette smoking or ST use (either) or dual-use use among the youth in 18 countries in the Western Pacific region [file epih-44-e2022103-Supplementary-2.docx]

**Supplementary Material 2.**  sex-wise weighted prevalence estimates of cigarette smoking or ST use (either) or dual-use use among the youth in 18 countries in the Western Pacific region

|  | boys | Girls | boys | Girls | boys | Girls |
| --- | --- | --- | --- | --- | --- | --- |
|  | Either | | Dual | | Cigarette smoking | |
| **Brunei Darussalam** | 13.3 (8.8, 17.8) | 2.9 (1.8, 4.0) | 0.2 (0.0, 0.4) | 0.0 (0.0, 0.1) | 12.5 (8.2, 16.7) | 2.8 (1.7, 4.0) |
| **Cambodia** | 2.7 (1.6, 3.8) | 1.0 (0.5, 1.6) |  | 0.0 (0.0, 0.1) | 1.5 (0.5, 2.5) | 0.3 (0.1, 0.6) |
| **Cook Islands** | 20.4 (20.4, 20.4) | 14.1 (14.1, 14.1) | 3.8 (3.8, 3.8) | 2.0 (2.0, 2.0) | 23.4 (23.4, 23.4) | 15.0 (15.0, 15.0) |
| **Fiji** | 16.5 (12.6, 20.5) | 8.3 (5.6, 11.0) | 0.7 (0.3, 1.1) | 0.4 (0.1, 0.7) | 16.6 (12.3, 20.9) | 7.8 (5.3, 10.3) |
| **Guam** | 11.3 (9.0, 13.5) | 9.5 (7.7, 11.3) | 6.2 (4.5,7.9) | 3.0 (1.8, 4.1) | 13.0 (10.9, 15.1) | 8.3 (6.5, 10.0) |
| **Kiribati** | 28.7 (25.7, 31.6) | 32.0 (28.3, 35.6) | 25.0 (20.7, 29.4) | 10.4 (8.2, 12.5) | 34.7 (29.6, 39.9) | 15.1 (12.2, 17.9) |
| **Lao PDR** | 15.5 (12.6, 18.5) | 4.2 (3.2, 5.2) | 1.2 (0.7,1.6) | 0.1 (0.0, 0.3) | 13.4 ((10.8, 16.0) | 2.0 (1.1, 3.0) |
| **Macao, China** | 7.3 (3.6, 11.0) | 4.1 (2.1, 6.0) | 0.2 (-0.1, 0.5) | 0.6 (-0.1, 1.3) | 6.0 (2.4, 9.5) | 3.5 (1.2, 5.9) |
| **Marshall Islands** | 22.1 (19.2, 25.0) | 17.8 (15.3, 20.2) | 23.3 (20.0, 26.6) | 5.9 (4.6, 7.2) | 35.7 (31.3, 40.0) | 11.3 (9.3, 13.2) |
| **Micronesia,** | 22.3 (20.2, 24.3) | 17.7 (15.9, 19.5) | 16.2 (14.1, 18.3) | 8.6 (7.1, 10.1) | 29.4 (26.7, 32.1) | 18.9 (16.9, 20.9) |
| **Mongolia** | 16.8 (13.7, 20.0) | 5.2 (4.1, 6.2) | 1.5 (0.9, 2.2) | 0.1 (0.0, 0.3) | 8.3 (6.2, 10.5) | 1.0 (0.5, 1.6) |
| **Niue** | 16.5 (6.4, 26.6) | 7.4 (2.9, 11.9) | 1.5 (-1.4, 4.4) | 1.1 (-1.0, 3.2) | 17.2 (5.9, 28.6) | 7.4 (2.9, 11.9) |
| **Palau** | 33.0 (28.5, 37.5) | 25.0 (21.6, 28.4) | 8.0 (5.5, 10.5) | 9.5 (7.2, 11.8) | 35.6 (30.9, 40.3) | 28.0 (24.3, 31.8) |
| **Papua New Guinea** | 28.9 (21.6, 36.1) | 20.7 (13.4, 27.9) | 10.0 (5.9, 14.0) | 3.9 (2.1, 5.7) | 34.1 (25.9, 42.4) | 15.7 (8.3, 23.1) |
| **Philippines -** | 21.1 (18.2, 24.1) | 7.6 (6.2, 9.1) | 1.3 (0.9,1.7) | 0.4 (0.0, 0.7) | 22.2 (19.1, 25.2) | 7.7 (5.8, 9.6) |
| **Samoa** | 21.7 (17.4, 26.0) | 6.6 (4.7, 8.5) | 1.0 (0.4, 1.5) |  | 21.1 (17.0, 25.3) | 5.0 (3.2, 6.8) |
| **Tuvalu** | 22.9 (16.9, 29.0) | 12.4 (8.5, 16.3) | 1.3 (-0.2, 2.9) | 0.2 (-0.2, 0.7) | 22.1 (15.5, 28.6) | 11.0 (7.0, 15.0) |
| **Vanuatu** | 27.6 (22.4, 32.7) | 15.9 (11.2, 20.5) | 2.6 (1.2, 3.9) | 1.6 (0.3, 2.8) | 28.1 (22.8, 33.5) | 14.7 (9.3, 20.2) |
