# Supplementary material for: Smokeless tobacco consumption and its association with tobacco control factors in the Western Pacific Region: results from the Global Youth Tobacco Survey 2015-2019
Source: Epidemiol Health. 2022 Nov 8;44:e2022103. doi: 10.4178/epih.e2022103 (PMC10185971; doi:10.4178/epih.e2022103)
Supplement: Supplementary Material 3 — Individual and country-level factors associated with ST use and dual-use by multi-level analyses after inclusion of parental smoking status in 12 countries of WHO WPR [file epih-44-e2022103-Supplementary-3.docx]

**Supplementary Material 3.**  Individual and country-level factors associated with ST use and dual-use by multi-level analyses after inclusion of parental smoking status in 12 countries of WHO WPR

|  |
| --- |

|  | ST use | | Dual-use | |
| --- | --- | --- | --- | --- |
|  | aOR (95% CI) | p-value | aOR (95% CI) | p-value |
| Individual-level factors | | | | |
| Sex |  |  |  |  |
| Boys | Ref. |  | Ref. |  |
| Girls | 0.74 (0.67, 0.82) | <0.001 | 0.43 (0.34, 0.60) | <0.001 |
| Age group | | | | |
| 11-13 | Ref. |  | Ref. |  |
| 14-15 | 1.28 (1.11, 1.47) | <0.001 | 2.50 (2.06, 3.28) | <0.001 |
| 16-18 | 1.85 (1.59, 2.15) | <0.001 | 4.31 (3.42, 5.44) | <0.001 |
| Parental smoking status |  |  |  |  |
| None/don’t know | Ref. |  | Ref. |  |
| Either parent | 1.23 (1.10, 1.37) |  | 1.47 (1.26, 1.73) | <0.001 |
| Both parents | 1.68 (1.44, 1.97) |  | 2.15 (1.74, 2.65) | <0.001 |
| Pro and anti-tobacco factors | | | | |
| Exposure to anti-tobacco messages on mass media | | | | |
| No |  |  | Ref. |  |
| Yes | 1.13 (1.03, 1.28) | 0.012 | 1.31 (1.13, 1.53) | 0.001 |
| Exposure to anti-tobacco messages at the events | | | | |
| No/did not attend |  |  | Ref. |  |
| Yes | 1.01 (0.91, 1.12) | 0.863 | 1.26 (1.08, 1.45) | 0.002 |
| Taught about the dangers of tobacco use | | | | |
| No/don’t know | Ref. |  | Ref. |  |
| Yes | 1.22 (1.10, 1.36) | <0.001 | 1.45 (1.24, 1.70) | <0.001 |
| Exposure to tobacco imagery on TV/movies | | | | |
| No/did not watch |  |  | Ref. |  |
| Yes | 1.16 (1.04, 1.29) | 0.006 | 1.13 (0.95, 1.32) | 0.115 |
| Exposure to tobacco advertisements | | | | |
| No/did not visit | Ref. |  | Ref. |  |
| Yes | 1.09 (0.98, 1.22) | 0.122 | 1.32 (1.14, 1.53) | <0.001 |
| Offered free tobacco products | | | | |
| No |  |  | Ref. |  |
| Yes | 1.74 (1.51, 1.99) | <0.001 | 2.6 (2.19,3.09) | <0.001 |
| Country-level factors | | | | |
| Year of survey | | | | |
| 2015-17 | Ref. |  | Ref. |  |
| 2018-19 | 3.66 (2.81, 4.76) | <0.001 | 4.57 (3.31, 6.30) | <0.001 |
| Country-income | | | | |
| Lower middle | Ref. |  | Ref. |  |
| Upper middle | 0.25 (0.18, 0.35) | <0.001 | 0.19 (0.12, 0.33) | <0.001 |
| High | 0.39 (0.23, 0.67) | 0.001 | 0.38 (0.18, 0.79) | 0.009 |
| MPOWER score | 0.76 (0.70, 0.81) | <0.001 | 0.55 (0.50, 0.60) | <0.001 |

Data on parental smoking was unavailable for 5 countries (Brunei, Lao PDR, Marshall Islands, Palau, Philippines). MPOWER scores were unavailable for Guam
